# The American and Colonial Journals: Anatomy and Physiology

**Published:** 1839-10

**Authors:** 


### II. THE AMERICAN AND COLONIAL JOURNALS.

### PHYSIOLOGY.

### *Contributions Illustrative of the Functions of the Cerebellum.*

By JOHN D. FISHER, M.D., of Boston.

THREE different functions have been attributed to the cerebellum by as many classes of writers. One class maintain the doctrine that this organ is the regulator of the movements of locomotion; a second, that it is the centre and source of sensation; and a third, that it is the organ of the instinct of reproduction. The advocates of each of these theories will read the following details with some degree of interest, and the followers of the founder of the new theory of cerebral physiology and of mental physiology, will not fail to summon some of them as proofs of their adopted doctrine.

CASE I. D. O. æt. forty-five. First seen a few hours after death, which took place from pneumonia, as was proved by dissection. On carefully examining penis (which, as was stated, appeared very small,) the prepuce was found covering the glans, and seemed as if it had been seldom or never retracted; in fact the glans was with difficulty made to pass through it, the aperture being so much contracted. When exposed it was pale, small, and pointed, and the urethra of exceedingly small caliber. All the parts of the organ resembled perfectly those of a boy not yet arrived at the age of puberty. The scrotum was soft and flabby; it contained no testicles, but it was thought that the spermatic cord could be felt at the upper part. An incision was then made, commencing at the inguinal ring and extending to the lower extremity of left side of scrotum. The skin, the dartos, and the tunica vaginalis, were of a natural appearance, but no testes nor any bodies of a glandular nature existed in the scrotum. In the upper part of the left tunica vaginalis, the spermatic cord was discovered extending into its cavity about half an inch, and terminating abruptly in a point of a semi-lunar shape. The cremaster muscle was seen extending in numerous small fibres beyond the terminus of the cord which spread themselves out upon the tunica vaginalis. The cord itself was much smaller than is usual in adults. The vas deferens was properly formed and nearly of natural size; its cavity terminated in a cul-de-sac at the end of the cord. The arteries and veins were exceedingly small, hardly distinguishable. The right side of the scrotum and the right spermatic cord differed in no respect from the left except that the latter extended to the bottom of the scrotum and turned upwards a quarter of an inch. So far as they were traced into the abdomen they presented no other peculiarities. Circumstances prevented examination of the *vesiculæ seminales;* it is therefore impossible to say whether they existed or not. From the perfect condition of the vas deferens, it is presumed they did exist, and might have been found.

The history of the individual, the absence of the testes, and other circumstances, having brought to mind the doctrines of phrenology in relation to the functions of the cerebellum, the examination was extended to the cranium and its contents, with a view to testing the truth of these doctrines. The size of the head was found by measurement to be as follows, viz. 22 inches in circumference from the middle of the forehead over the crucial ridge of occiput; 16 inches from the orifice of one ear to that of the other over the highest point; 6 inches from mastoid process to mastoid process; and 8 inches from ear to ear. The head, therefore, was a large one. On opening cranium and removing brain, it was found to be in a healthy condition, and of large size—but the relative proportion of the cerebrum to the cerebellum was strikingly unnatural; the latter being comparatively exceedingly small. Upon weighing the encephalon, comprising the cerebrum and cerebellum on the day following, it was found to weigh 51½ ounces—or 3 pounds 3½ ounces avoirdupois. The cerebrum alone weighed 47 ounces. The cerebellum alone weighed 4½ ounces. The weight of the cerebellum to that of the cerebrum was, therefore, as 1 to 10½ within a fraction. The cerebellum measured in its transverse diameter 4 inches, in its antero-posterior diameter 2½ inches, and in thickness 1½ inch. According to Meckel and others the average weight of the cerebrum and cerebellum united is 3 pounds, and the weight of the cerebellum to that of the cerebrum as 1 to 7, or 1 to 8. Its usual measurement being in its transverse diameter 4 inches, in its antero-posterior diameter 2½ inches, and in thickness 2½ inches. The cerebellum, therefore, in this person was one third less in size and weight than is naturally the case in an adult male—and was the exact weight of that of a female child six years old, who died, and whose cranium was dissected at the same period.

The history of the patient who forms the subject of the preceding case, furnished by a near relative, is of much physiological interest. He was born in 1791, and was, therefore, 45 years of age at the time of his death. The late Dr. Warren discovered the deficiency of testes soon after birth, and observed that he would probably prove to be a natural eunuch. He grew up to the age of puberty without exhibiting any peculiarities distinguishing him from his fellows, except the non-appearance of the testicles. From the age of puberty to the age of twenty-five, and even to the day of his death, he presented the following peculiarities. His voice remained unchanged in its tone, which was decidedly effeminate. He was fond of music, and sung with much taste and effect, but always in treble and in concert with females. After the age of 25, however, it became grave, and he could no longer accompany female voices with ease. He had no beard, and was never known to shave. He never exhibited any amorous propensities or desire for female society. Although of a social disposition, he was very shy in company with females of his own age, and always approached them with evident timidity. He was extremely guarded in his expressions before ladies, and often reprimanded his associates for using in their presence language in the least degree expressive of an indelicate or amorous sentiment. When about 21 years of age he became acquainted with a number of young men fond of pleasure and frolic, and by degrees acquired a taste for the inebriating cup, but during the many scenes of dissipation in which he participated, he was never known to visit a house of ill-fame, or to address any of the numerous ladies of pleasure who walked the street. In short he was, as his mother expressed herself, "a virgin in feeling and conduct to the day of his death."

CASE II. T. P. B , æt. forty-one, on the 29th June, 1836, was a passenger in one of the trains of railroad cars which unfortunately came in collision on the track between Boston and Providence. While seated on a front seat of a car, with his back directed towards the engine, Mr. B. noticed that a sudden effort was made, by means of the "breaker," to arrest the progress of the cars. On observing this, he arose from his seat, and thrust his head out of the window to ascertain the meaning of this operation. At this moment, and while the back part of his head and neck were opposite the edge of the window-frame, the two trains came in collision with tremendous and fearful violence. The consequences of this accident were, that the cars were broken into many fragments, and that most of the passengers who occupied them were thrown out and seriously injured. Mr. B.'s head and neck were brought up against the edge of the window-frame with great force, and he himself was projected to a distance upon the ground, where he remained for sometime in a state of insensibility. When he was first lifted up, it was thought by his fellow-passengers that he was fatally wounded—that his skull was fractured, or the bones of his neck dislocated. He, however, regained his intelligence, and was soon conveyed to his dwelling in a carriage. On visiting him one hour after the accident, I found him in his bed, suffering great pain in the occipital portion of his head and upper part of the neck. He was lying on his back, unable to rotate his head on the pillow, or to move from a horizontal position. Every attempt to move himself was attended by excruciating pain, and he would not allow others to move him for fear of suffering.

On examination, some blood was found on his face, and a flesh-wound of minor importance was discovered in the integuments covering the left mastoid process, and the inferior portion of the occipital bone. These parts were somewhat swollen and tender, as were the integuments and muscles of the neck. No indications of fracture of the cranium existed, nor could any dislocation or fracture of the cervical vertebræ be detected.

For two or three days and nights he obtained little or no sleep, and could not be moved from his horizontal position. The antiphlogistic treatment was continued for some days, when he was so much relieved, that he could, by much care and cautious effort, raise himself from his bed. To do this, he was first obliged to turn cautiously from his back upon his left side, and then raise himself to the sitting posture by the aid of his left elbow. This was the only method he could adopt to raise himself from his bed without rotating or moving his head, which movement was constantly accompanied by extreme suffering. On the second day after the accident, he complained of a numbness in his right arm, and experienced a difficulty in passing his urine. The contractile power of the bladder seemed to have been diminished, and he was compelled to resort to artificial means to evacuate the organ. In the course of two weeks he was able to leave his bed and to walk into the street. And now another interesting symptom was manifested. As he walked about the house and in the street, he observed a singular appearance in the objects about him. Near objects seemed to him to be at a distance, and he felt as if he was much elevated above them. While walking, the street seemed to be interminable in length; and when standing by and conversing with a person of his own height, he experienced the feeling that he was vastly the tallest, and that he was actually looking down upon him during the conversation, yet all objects appeared natural in colour, size, and proportion. Between the fourth and fifth week after his injury, he made the discovery that he had lost the desire and physical power for sexual intercourse, and that no amorous sentiment or the approach of a female could excite it; and he was of opinion that the amative instinct and sexual desire had ceased to exist from the time he was wounded. These symptoms, which were the prominent and peculiar ones of the case, were exceedingly troublesome, and were for a long time combated by local bleeding, blistering, and other remedies. The bladder gradually recovered its power, and the aberration of visual perception was by degrees corrected, so that in the course of four months, Mr. B. was enabled to make water freely and naturally, and to view objects in relation to himself as he was accustomed to see them previous to his injury. He was still, however, unable to rotate his head, and when he attempted to do so, he heard a sort of grating noise, which evidently arose from the deranged action of the vertebræ of the neck, or of the ligaments or muscles attached to them. The numbness of the right arm still continued, and the limb had decreased in size, its circumference being considerably less than that of the left arm. The instinct of generation, for which he was particularly distinguished while in health, was still dead, and the idea of its total annihilation gave him much uneasiness. The trouble in moving the head and the numbness of the arm continued for some months, and the generative function remained completely silenced, according to B.'s own report, until the last summer, and is even now (Dec. 18, 1838,) but partially restored. The mental powers of this patient, particularly his memory of events, were for a time seriously affected, and his decision, courage, and resolution enfeebled. In these respects, however, as in most others, the individual now enjoys his accustomed health and strength.

Case iii. (By Dr. Whittimore). "Mr.——, ætat. seventy-three, has been married about forty years, has had eleven children, ten of whom, as also his wife, are still living. Mr.—— worked alternately as a shoemaker and farmer; frame large, and general appearance that of robust health. Soon after marriage he began to complain of dizziness and noises in head, to which he was more or less subject until his death. About four years ago he experienced, on rising from bed, for three or four mornings in succession, excruciating pain in the head, which was followed by a sensation as if something had given way in left side of head with an audible crack, such as to lead him to enquire if the bystanders did not hear the sound, and was surprised to find they did not. After this, he became partially deaf in left ear, and the dizziness increased. During these dizzy turns, he was obliged to catch hold of the nearest object to keep from falling, and at such times everything seemed to be whirling about like wheels, with a motion always from right to left; these symptoms were for the most part attended with great heat and pain about head, and with redness of scalp. During the severity of suffering he was at times delirious. Most relief was gained by cupping nape of neck and very little from other means.

"Two years ago had hemiplegia of right side, and has had two other attacks since, all slight. Since the occurrence of these he has had a *morbid salacity*, which continued with little intermission, and increased by degrees till three months ago, and then gradually subsided, so that the desire became imperious only once or twice during the night without ability to gratify it, owing to imperfect erection, and for the last year there has been no seminal emission. This lustful feeling aggravated all his other sufferings, and destroyed most of his comfort. Such is the account of the patient, as given by a particular friend; but the patient during his sickness, expressed himself strongly on the subject of this revived sexual propensity, and declared that the desire was felt many times during the day and night, and was scarcely diminished by the frequent attempts he made to gratify it. For the last year, he has been decidedly growing worse, both in body and mind—*mind in a state of imbecility*. Early in summer he had an *epileptic* paroxysm, and within a few weeks before his death had a second. For the last five months was occasionally delirious—screaming as if frightened, and sometimes as if in pain—was afterwards unconscious of what had happened. He died on the 18*th September*, 1835, having been in a state of stupor for some days.

"On the following day the head was examined. The membranes of the brain presented some morbid appearances, such as very strong adhesion of the *dura mater* to the skull; thickening with white spots in the arachnoid and a large quantity of serous fluid in the pia mater; arteries undergoing ossification. The brain was healthy, except for the disease to be described. The cerebellum being removed for examination, the right lobe was found to be of its full size; the left about one fifth smaller, and the greater part of its under surface in a *remarkably* collapsed state—*hollowed in* as an organ usually appears *externally*, when there has been any very great loss of substance within. In one place, to the extent of about three lines square, the disease beneath had penetrated quite to the surface, and over this the *pia mater* contained a considerable quantity of dull yellowish serum, elevating the *arachnoid;* otherwise the surface of this lobe of the cerebellum looked well, retaining its integrity, natural colour, and consistence. An incision having then been made through the collapsed portion to the centre of the lobe, and another to cross it, it was fully demonstrated that the whole substance of the organ below the *crus cerebelli* was destroyed, and all traces of it gone, except a line or two in thickness, of the very surface which served as *parietes* to what may be called the cavity. The sides of the cavity were in contact, and were connected here and there by a very soft delicate tissue of a light rusty brown colour, and were separated by the slightest force. The same sort of substance lined the cavity, appearing in some parts a mere discoloration of the inner surface; in others like a distinct tissue, passing by invisible degrees into the *pia mater*, where it dips down between the lobes of the cerebellum. The cavity very probably contained some serum, but if so, it had escaped before it was laid open. The crus cerebelli had a dull, somewhat opaque yellowish colour, and was considerably firmer than natural, especially towards its under surface, where it bounded the cavity. This surface was also somewhat irregular, as if a small portion of it had been destroyed by disease. On cutting through its substance, there was found a coagulum of dark-coloured blood of the size of a duck-shot; the remainder of the cerebellum was healthy."

In this case, we have a remarkable pathological proof of a relation existing between the cerebellum and the instinct of reproduction, and the revival of the instinct and powers of propagation (which had for years been extinguished,) taking place on the accession of a disease of the cerebellum, and continuing active until the organ began to lose its firmness of texture, and to undergo disorganization, is strong confirmation of the evidence furnished by the two preceding cases, that this part of the brain is the source and centre of the instinct.

*American Journal of Med. Sciences. Feb.* 1839.

*On the Physiology of the Iris.* By James Bolton, m.d. of Baltimore.

By what means are the contraction and dilatation of the pupil immediately produced? The only modes of explanation adopted at present are the two following: the one that it is an erectile tissue, and the other that it is muscular, and composed of two sets of fibres, one radiating and the other circular. The first mode I do not think at all satisfactory, and its advocates are far less numerous than those of the second. The motions of the iris are by far too rapid to be accounted for in this way, and no contrivance has ever been discovered by which its sudden injection with blood and the sudden withdrawal of the blood can be accomplished. The dilatation of the pupil, when the head and face are gorged with blood, as in apoplexy, and the effects of injuries of the brain and its contraction when these are relieved by bloodletting, I consider an insuperable objection to this theory. Brodie has frequently observed the dilated pupils to contract after the abstraction of blood in cases of compression of the brain, and to dilate again as soon as the immediate effect of bloodletting had ceased.

We consider the point settled, that the iris contracts by a sphincter muscle. The only point then remaining to be settled is how the dilatation is produced. The advocates of the muscularity of the iris have generally considered that the dilatation must necessarily be produced by the same cause as that which produces contraction. It is true that many distinguished anatomists have admitted a set of radiating fibres, but we should be cautious how we attribute muscularity to any organ merely because it is fibrous. The cellular tissue may be drawn out into fibres, fasciæ: tendons, nerves, &c. are fibrous, but they are not therefore muscular. But, besides having a fibrous structure, the iris dilates: and these two circumstances, so far as they go, show that it possesses a set of radiating muscular fibres. Let us apply this theory to the phenomena. In amaurosis the pupil is permanently dilated. Now, can it be conceived that a muscle can remain for twenty, thirty, or forty years in a state of constant contraction? The idea is totally inadmissible. This disease is a paralysis of the nerve of vision, and in no way affects the nerves of the iris; we shall presently see why then the iris is at all affected in it. Belladonna and stramonium also cause dilatation of the pupil. This effect, when they are applied externally, is no doubt produced by their action being transmitted by those branches of the first branch of the fifth pair, which supply the conjunctiva and eyelids, to the ciliary branches of the same nerve. Do these substances, therefore, paralyze the circular fibres, without in the least degree affecting the radiating, and these latter then act constantly? I cannot conceive it at all probable that these powerful narcotics would thus distinguish between these muscles, when both (if they do exist) are supplied by the same nerves. I know this latter point also is contested, but there is no proof adduced of a different arrangement. It is merely an invention to explain a difficulty which can be much better explained in a different way. Besides, the theories of Magendie and Bellingeri, who contend for a different supply of nerves for each muscle, are directly opposed to each other as to which nerve goes to the contractor and which to the dilator. The iris has not been known to dilate from the galvanic stimulus. From all these circumstances we conclude that the idea of a dilator muscle in the iris is incompatible with some of its most important phenomena. Now, if we admit the radiating fibres to be elastic, we have an easy and satisfactory explanation of all the phenomena. In amaurosis the optic nerve no longer receives any impression, and none consequently is transmitted to the iris. The sphincter is therefore passive, and gives up the iris entirely to the power of the elastic fibres which dilate it. Expansion of the iris from belladonna is caused by a direct paralysis of the ciliary nerves, while the nerve of vision is not affected. A difficulty here presents itself, how the pupil is kept in a state of contraction for so many hours each day, if this be produced by a muscle. This difficulty, however, is readily explained. The sphincter is relieved from a state of constant contraction, 1st, by our passing through different shades of light, causing slight contractions and dilatations; 2d, by winking, which we do involuntarily every few seconds. When the eyelids are closed the pupil dilates, and on opening them it instantly contracts. This is the principal mode of resting the sphincter, and shows that the action of winking possesses a highly important use, besides that usually ascribed to it. That this momentary rest is sufficient is proved by analogy. The wings of some birds and insects move several thousand times in a minute, and yet the intervals between the contractions are sufficient to rest the muscles. 3d. During sleep, this muscle, together with the rest of the whole muscular system, rests and renews its strength. The eminent anatomist, Dr. Wistar, taught the same doctrine as that which I have advocated.

The following experiments will, I think, prove incontestibly the theory here supported. If a fresh eye be cut through parallel to the iris, and a little way behind it, and the front half be immersed in water, the elasticity of the iris may be proved by stretching it toward the pupil, and it will be found to resist and to return to its former position immediately on being relieved from this state of tension. The second experiment is still more conclusive. It occurred to me that if the mechanism of the iris were such as I have been endeavouring to prove, I might weary the contractor muscle by preventing the eye from winking for a considerable time. Accordingly, having a bright lamp placed about a foot from my left eye, I kept the eyelids open with the thumb and forefinger of the left hand· Then holding a mirror a few inches from the eye, I closely watched the iris. In a few seconds there was a smarting sensation, attended by involuntary attempts to wink. Immediately the pupil expanded and then quickly contracted. For two or three minutes alternate contractions and expansions took place incessantly, and then the pupil remained for a few seconds widely expanded, giving to the eye an amaurotic appearance. A partial contraction then took place, followed by an immediate expansion. In two or three seconds, contraction again took place, closing the pupil about as much as at the commencement of the experiment. The contractions and expansions were again incessant for about a minute, when the experiment was concluded. I have repeated this experiment several times on my own eyes and others, and always with similar results.

*American Medical Intelligencer.* 1838.

---

*On the Pulsations of the Fœtal Heart and the Umbilical Cord in Utero.* By Prof. Dunglison, of Philadelphia.

[It is well known that Dr. Hamilton, of Edinburgh, in his work on Practical Midwifery, has asserted that the fœtal pulse was never felt by him above 60, previous to respiration, whether examined in the region of the heart, or in the umbilical cord. This statement, so utterly at variance with the experience of all auscultators, is, probably, explained satisfactorily in the following extracts.]

To us, who have had so many opportunities of hearing the pulsations of the fœtal heart *in utero*, it would seem in the highest degree astonishing that an individual of Prof. Hamilton's experience could question the fact, were it not that he admits he has never attempted to verify or disprove it. It would be still stranger, however, were his observations accurate, that whilst the fœtal heart has been generally counted beating 130 or 140 times in a minute, the umbilical cord should not pulsate more than 50 or 60 times. Although satisfied of the inaccuracy of his deductions, we have repeated our own observations. Not many cases occur in which there is a good opportunity for examining this point. The best are those in which turning becomes necessary ; but the practitioner is then so anxious to relieve his patient from suffering, and so absorbed with the operation, that the opportunity can rarely be embraced. Where the cord protrudes, this can be accomplished ; and at times after the child is born. In these cases we can confirm the statement of our able friend, Dr. Meigs, in a note on this matter with which he has recently favoured us: who says, that he has very carefully observed the pulsations of the umbilical cord, while the ear was applied upon the region of the heart ; and in every instance the pulsations were isochronous. The truth would seem to be, that in the cases examined by Prof. Hamilton, owing to the influence of the parturient efforts on the function of innervation, and through it on the circulation, the pulsations of the fœtal heart were unusually depressed; but in every case he would, doubtless, have found them isochronous with those of the umbilical cord, had he made the trial. It is obvious, indeed, that they must be so, seeing that the umbilical arteries are but a part of the circulatory apparatus of the fœtus. In a case observed by Dr. Vedder, an intelligent and zealous resident physician at the Philadelphia Hospital, whose name often occurs in these pages, and whose attention was directed to this subject at our request, he noticed that while the uterus was quiescent the pulsations numbered 140 per minute; but that immediately succeeding a pain, they were only 96, and then gradually rose to 140. After delivery, the cord and fœtal heart beat respectively 134 in the minute. The observations of Prof. Hamilton should not, therefore, be permitted to weigh with the observer. They are imperfect, inasmuch as the pulsations of the fœtal heart were not attended to whilst he numbered the beats of the cord; and, consequently, they in no respect conflict with the observations of almost every other obstetrical physiologist, that the sounds actually heard during pregnancy, referred to the fœtal heart, are owing to the pulsations of that organ.

In one of our recent German journals (*Neue Zeitschr. für Geburts.* B. vi. s. i.) we find an interesting communication elucidative of the matter by D. Von Hoefft, of St. Petersburg, detailing certain observations made on pregnant females in the Imperial Lying-in Hospital (*der Kaiserlichen Gebäranstalt,*) of St. Petersburg. Some of the conclusions of Dr. Hoefft are as follows:

*a.* In regard to the circulation between the mother and the child. From the great difference between the number of beats of the maternal heart and that of the fœtus, it is clear and evident that no community of circulation exists, as the pulse of the mother is ordinarily a third, or a half, and in rare cases, even two thirds less numerous than that of the child. Hence it is manifest, that after the death or cessation of the circulation, of the mother, the fœtus may continue to live for some time in utero, as experience has shown. Auscultation, consequently, has proved *biologically* what Hunter's preparations had exhibited *anatomically.*

*b.* The influence of uterine contraction on the circulation of the fœtus is exhibited in the most marked manner. During slight pains, the pulsations of the fœtus continue; but during more violent contractions, especially after the discharge of the waters, they are wholly interrupted, so that we may presume the fœtus to be in a state of temporary asphyxia in the last periods of labour, and that great danger may threaten the child if the pains continue for a long time without interruption. *American Med. Intelligencer. Jan. and April,* 1839.

